# Erectile dysfunction: role of computed tomography cavernosography in the diagnosis and treatment planning of venous leak

**DOI:** 10.1186/s42155-023-00403-9

**Published:** 2023-11-17

**Authors:** Hanno Hoppe, Dominique Hirschle, Martin Christian Schumacher, Heinz Schönhofen, Michael Glenck, Christoph Kalka, Torsten Willenberg, Sebastian Sixt, Dominik Müller, Andreas Gutzeit, Andreas Christe, Vignes Mohan, Nicolas Diehm

**Affiliations:** 1 University of Bern, Bern, Switzerland; 2 Department of Health Sciences and Medicine, University of Lucerne, Lucerne, Switzerland; 3 Microtherapy Center Bern, Lindenhofspital, Bern, Switzerland; 4 Campus Stiftung Lindenhof Bern, Bern, Switzerland; 5 Department of Urology, Hirslanden Clinic Aarau, Aarau, Switzerland; 6 Radiology Center Baden, Baden, Switzerland; 7 Vascular Institute Central Switzerland, Aarau, Switzerland; 8 Vascular Center Bern, Lindenhofspital, Bern, Switzerland; 9 Vascular Center Biel, Biel, Switzerland; 10 Diagnostic, Interventional and Pediatric Radiology, Inselspital, Bern University Hospital, Bern, Switzerland; 11 University of Applied Sciences Furtwangen, Villingen-Schwenningen, Germany

**Keywords:** Erectile dysfunction, Venous leak, Venous incompetence, Computed tomography cavernosography

## Abstract

**Background:**

Venous leak appears to be the most common cause of vasculogenic erectile dysfunction (ED), which can be treated with venous embolization. Traditionally, conventional cavernosography was used for the diagnosis and treatment planning of venous leak. Recently, computed tomography (CT) cavernosography was introduced as a novel cross-sectional imaging method proposed to be advantageous over conventional cavernosography. We created a novel management algorithm for diagnosing venous leak including CT cavernosography as an imaging modality. In order to provide a broader basis for our management algorithm, a systematic literature review was conducted.

**Main body:**

In this article we systematically review relevant literature on using CT cavernosography for the diagnosis and treatment planning in ED patients with venous leak following the PRISMA selection process. Nine full-text articles were included in the review and assigned a level of evidence grade (all grade II). Two studies (2/9) compared the results of conventional cavernosography with those of CT cavernosography which was superior for site-specific venous leak identification (19.4% vs. 100%, respectively). CT cavernosography is a more detailed imaging method that is faster to perform, exposes the patient to less radiation, and requires less contrast material. In one study (1/9), CT cavernosography was used for diagnostic purposes only. Eight studies (8/9) cover both, diagnostic imaging and treatment planning including embolization (1/9) and sclerotherapy (2/9) of venous leak in patients with venogenic ED. Three studies (3/9) describe anatomical venous leak classifications that were established based on CT cavernosography findings for accurate mapping of superficial and/or deep venous leak and identification of mixed or more complex forms of venous leak present in up to 84% of patients. In addition to treatment planning, one study (1/9) used CT cavernosography also for follow-up imaging post treatment.

**Conclusion:**

CT cavernosography is superior to conventional cavernosography for diagnosis and treatment planning in patients with ED caused by venous leak (grade II levels of evidence). Consequently, CT cavernosography should be included in management algorithms for ED patients with suspected venous leak.

## Background

Erectile dysfunction (ED) is a male sexual dysfunction that is defined as the persistent inability to achieve and maintain a sufficient erection for satisfactory sexual intercourse [1]. Penile erection requires sufficient penile vasculature, relaxation of smooth muscle cells in the corpora cavernosa and high intracavernosal blood flow with sufficient occlusion of the efferent veins. ED may have a psychogenic, vasculogenic, endocrine, neurogenic, structural or iatrogenic etiology. The proportion of men with ED increases from 12.4% at age 40 to 46.4% at ages 60–69 [2]. This demonstrates that the main group affected by moderate to severe ED is predominantly individuals aged 60–69 years. The incidence of ED has also increased over time. In 1995, 152 million men were affected, and according to studies, by 2025, this number will have increased to 322 million [3]. This increase is mainly due to the growing proportion of elderly people in the population [4, 5]

Recently, ED treatment has evolved considerably and has been revolutionized by the introduction of phosphodiesterase-5 inhibitors (PDE5i), which are considered the first-line therapy for ED. However, up to 50% of patients on PDE5is still have insufficient erections for intercourse or experience relevant side effects [6]. As previously reported, a vascular etiology is highly likely in ED patients nonresponsive to PDE5is [7]. Basically, vascular pathologies were reported to account for more than 40% of all ED causes [8].

The main arterial supply of the penis is usually the internal pudendal artery that originates from the anterior division of the internal iliac artery. It supplies the dorsal penile artery, cavernosal artery, and bulbo-urethral artery (variations may occur) [9]. The dorsal penile artery supplies the glans penis and distal corpus spongiosum. The cavernosal artery supplies blood to the corpora cavernosa and helicine arteries acting as resistance arteries. The bulbo-urethral artery supplies the urethra and proximal corpus spongiosum [10].

Penile venous drainage consists of superficial, intermediate and deep veins (variations may occur). Superficial veins mainly drain into external pudendal veins. The intermediate veins, including the deep dorsal veins, drain blood into the prostatic venous plexus and internal pudendal vein. Deep veins, including the cavernosal, bulbo-urethral and crural veins, drain blood into the internal pudendal and periprostatic veins. Emissary veins drain the subtunical venular plexus into both intermediate and deep veins [11].

During penile erection, parasympathetic nerves are activated and release nitric oxide (NO). Subsequently, vascular resistance decreases and blood flow through the cavernosal and helicine arteries increases. Consequently, blood accumulates in the sinusoids, and intracavernosal pressure increases. Simultaneously, veins restrict their outflow through compression of subtunical veins against the tunica albuginea as a veno-occlusive mechanism.

The most common causes of vasculogenic ED are venous leak and arterial obstruction.

Venogenic ED appears to be the most common cause of vasculogenic ED [11, 12]. Rajfer et al. even reported that 86% of venous leaks occur in patients with organic ED [13]. Venogenic ED is caused by incomplete relaxation of cavernosal smooth muscle cells during arterial inflow and subsequent failure to occlude penile venous outflow tracts (venous leak). The exact underlying etiology of venogenic ED is not yet fully understood. As previously reported, venogenic ED may also be related to age- or injury-related changes in the tunica albuginea, cavernosal smooth muscle dysfunction due to structural alterations, excessive adrenergic input or shunts that may have developed during previous priapism episodes and subsequent repair [14]. Potential risk factors for the development of venogenic erectile dysfunction may be age, diabetes, prostatectomy, pelvic radiation, and androgen deprivation therapy. A venous leak can occur in a man of any age. Especially in younger patients, site-specific congenital, posttraumatic, or post inflammatory venous leaks may occur.

When working up patients with suspected vasculogenic ED, at first penile duplex sonography is performed after an intra cavernosal injection of alprostadil for pharmacological stimulation of a penile erection. An increased end-diastolic velocity (EDV > 5 cm/s) is indicative of venous leak [9]. In addition to penile duplex sonography, cavernosometry and/or cavernosography were traditionally used as complementary modalities for diagnosing venogenic ED [9, 15, 16]. Cavernosometry is performed by injecting normal saline solution into the corpora cavernosa at varying flow rates and simultaneously measuring intracavernosal pressure [17]. Cavernosography is conducted for confirmation of venous leak using fluoroscopically guided intracavernosal injections of contrast medium under radiographic imaging at different angulations [18]. However, cavernosography may have limitations, such as prolonged examination times, high radiation exposure, and the use of significant amounts of highly concentrated contrast material. Furthermore, it is not a cross-sectional imaging method with disruptive superimposition of surrounding anatomical structures, rendering site-specific visualization of venous leak difficult [19]. Computed tomography (CT) cavernosography is a novel cross-sectional imaging method that is proposed to be advantageous for diagnosis and treatment planning in terms of site-specific venous leak visualization and classification.

**Fig. 1:**
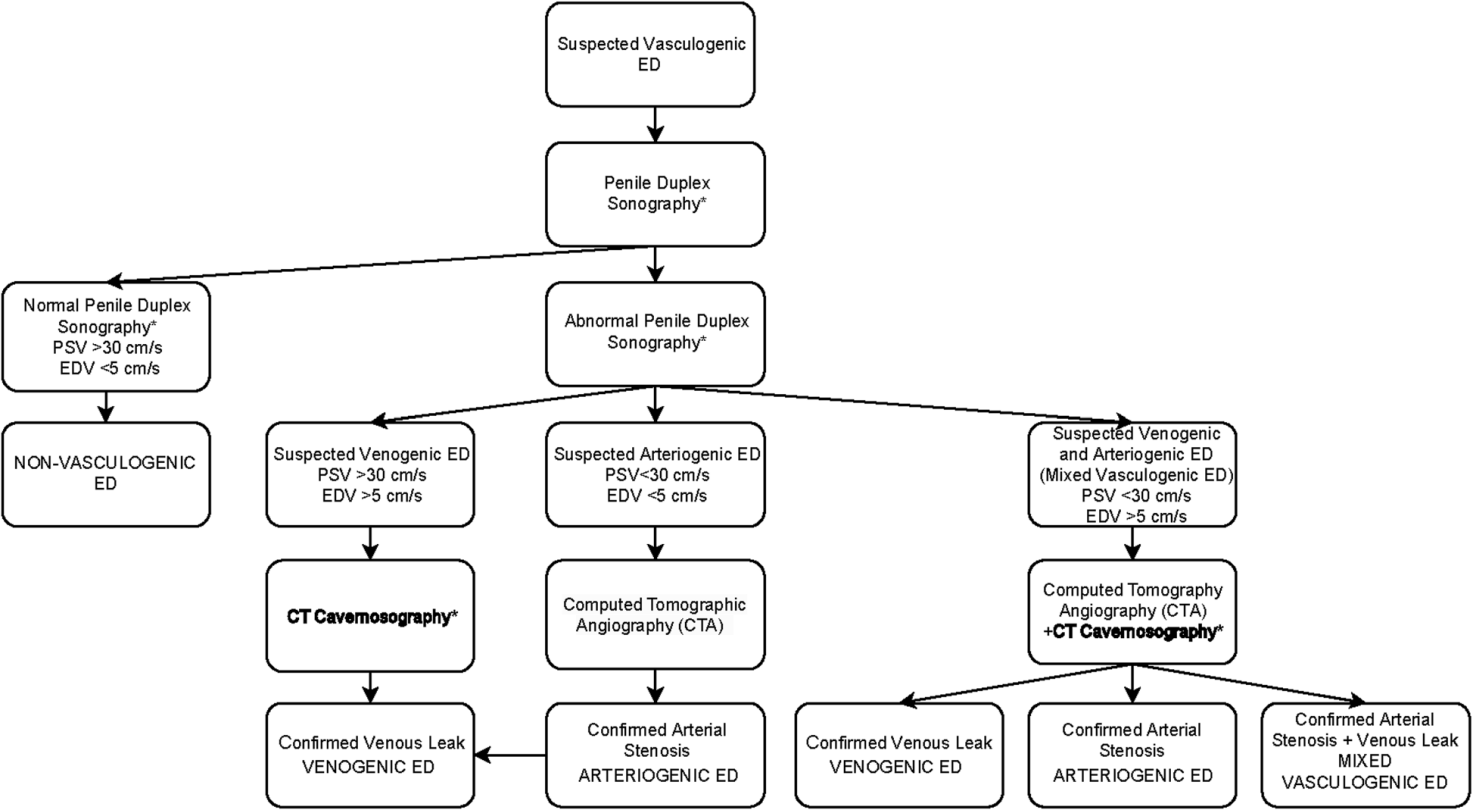
Suggested diagnostic management algorithm for patients with vasculogenic erectile dysfunction including CT cavernosography (bold) for diagnosing patients with venogenic erectile dysfunction (ED – erectile dysfunction, PSV – peak systolic velocity, EDV – end diastolic velocity, *—post intracavernosal injection of alprostadil)

We created a novel diagnostic management algorithm for diagnosing vasculogenic ED including CT cavernosography as an imaging modality if venogenic ED is suspected on penile duplex sonography (Fig. 1). If arteriogenic ED is suspected on penile duplex sonography (PSV < 30 cm/s, EDV < 5 cm/s), CT angiography (CTA) should be performed as an additional imaging modality [20, 21]. CT cavernosography and CTA may also be used for clarification if mixed vasculogenic (arteriogenic and venogenic) is suspected on penile duplex sonography (PSV < 30 cm/s, EDV > 5 cm/s). In order to provide broader support for our management algorithm including CT cavernosography, a systematic literature review was conducted.

## Main text

### Methods

A systematic literature review was performed following the PRISMA (Preferred Reporting Items for Systematic reviews and Meta-Analyses) selection process in order to investigate the role of CT cavernosography in the diagnosis and treatment planning of venogenic ED [22]. Therefore, PubMed, Web of Science, Scopus and Google Scholar databases were searched from their inceptions as of July 2023. Search terms included computed tomography cavernosography and erectile dysfunction. Exclusion criteria were recurrent articles from the same authors, abstracts and not full-text articles, review articles and case reports, articles written in other languages, and articles not consistent to the purposes of our research. No studies were excluded based on year of publication. Each full-text article was reviewed by two authors (DH and HH) to confirm that the eligibility criteria were met. Selected studies were evaluated and assigned a level of evidence grade based on adaptations from existing guidelines (Table 1) [23, 24].

**Table 1:** Levels of Evidence Adapted from the American Society of Plastic Surgeons and Johns Hopkins nursing evidence-based practice: Models and Guidelines ([23]; Dang and Dearholt 2017)

Level	Description
I	High quality prospective randomized controlled trials, cohort studies with adequate power or systematic review of these studies
II	Lower quality prospective cohort, retrospective cohort study, randomized controlled trials with untreated controls, or a systematic review/meta-analysis of these studies
III	Case–control study or systematic review/meta-analysis of these studies
IV	Case Series, consensus statements, society guidelines, practice guidelines
V	Expert Opinion: case report, clinical example, narrative reviews

### Results

**Fig. 2:**
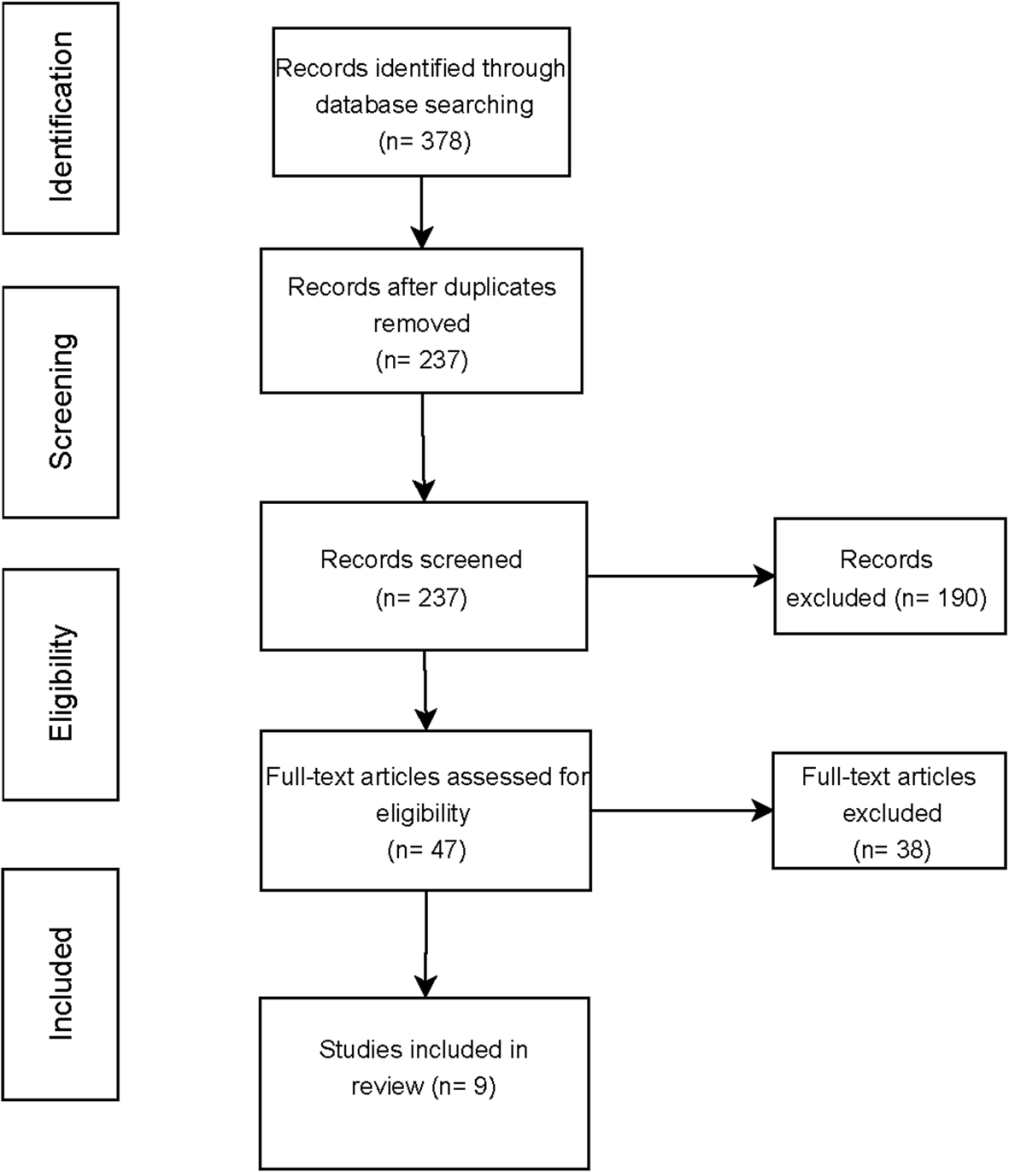
Flow diagram demonstrating the PRISMA search strategy: Of the 237 records screened, 9 studies were ultimately included in the systematic review

A total of 378 records were identified through database searching (Fig. 2). After removal of 141 records, 237 records were screened. Thereof, 190 records were excluded. Eventually, 47 full-text articles were assessed for eligibility, and 38 full-text articles had to be excluded because they were not consistent to the purposes of our research. Ultimately, 9 full-text articles were selected for review, all assigned a level of evidence grade II. A summary of these key studies (*n* = 9) on computed tomography cavernosography for diagnosis and treatment planning of venogenic erectile dysfunction with level of evidence designation is demonstrated in Table 2.

**Table 2:** Summary table of key studies (*n* = 9) on computed tomography cavernosography for diagnosis and treatment planning of venogenic erectile dysfunction with a level of evidence designation. Levels of evidence are defined using the grading system adapted from the American Society of Plastic Surgeons and Johns Hopkins nursing evidence-based practice: Models and Guidelines ([23]; Dang and Dearholt 2017)—(see Table 1)

Authors	Year	Title	Number of patients	Study design	Key points, data, summary	Level of Evidence
Ghafoori et al. [25]	2010	CT Cavernosography: A New Method for Evaluating Venous Incompetence in Impotent Patients	67	Prospective	Initial report on use of CT cavernosography for diagnosing venous leak in patients with erectile dysfunction and treatment planning	II
Kawanishi et al. [26]	2011	Three-dimensional CT cavernosography: reconsidering venous ligation surgery on the basis of the modern technology	55	Prospective	Using diagnostic CT cavernosography, site-specific venous leak identification was superior to simulated conventional cavernosography images with maximum intensity projection (100% vs.19.4%)	II
Virag et al. [27]	2011	New classification of anomalous venous drainage using caverno-computed tomography in men with erectile dysfunction	38	Prospective	Development of an anatomical classification allowing for differentiation between deep and/or superficial venous leakage important for diagnosis and treatment planning	II
Xu et al. [19]	2017	Comprehensive assessment of cavernosography with 320-row dynamic volume CT versus conventional cavernosography in erectile dysfunction patients caused by venous leakage	174	Prospective	Diagnosing venous leakage with CT cavernosography and conventional cavernosography (control group) and anatomical classification for venous leakage (superficial, deep, mixed, and other) for treatment planning	II
Herwig et al. [28]	2017	CT Cavernosography and Penile Venous Leak	49	Retrospective	CT cavernosography was successfully used in all patients with suspected venous leakage for diagnosis and endovascular treatment planning (82% reported clinical success rate of treatment)	II
Ye et al. [11]	2018	Computed tomography cavernosography combined with volume rendering to observe venous leakage in young patients with erectile dysfunction	186	Prospective	CT cavernosography for diagnosing venous leakage and classification as deep dorsal vein, prostatic venous plexus, crural vein, or complex (84.2%) as an important basis for appropriate treatment planning (endovascular embolization vs. surgical ligation)	II
Sussman et al. [29]	2020	Ultrasonography after pharmacological stimulation of erection for the diagnosis and therapeutic follow-up of erectile dysfunction due to cavernovenous leakage	50	Prospective	Comparison of Duplex sonography and CT cavernosography is superior for diagnosing venous leak demonstrating a low negative predictive value (47%) of duplex sonography recommending CT cavernosography also for treatment planning	II
Allaire et al. [30]	2021	Erectile Dysfunction Resistant to Medical Treatment Caused by Cavernovenous Leakage: An Innovative Surgical Approach Combining Pre-operative Work Up, Embolisation, and Open Surgery	45	Retrospective	CT cavernosography for pre-operative treatment planning of endovascular embolization and also for 3-months follow up (73% reported clinical success rate of treatment)	II
Diehm et al. [31]	2023	Venous Leak Embolization in Patients with Venogenic Erectile Dysfunction via Deep Dorsal Penile Vein Access: Safety and Early Efficacy	50	Retrospective	Diagnostic use of CT cavernosography in all patients for endovascular treatment planning of target vein embolization via penile venous access (68% reported clinical success rate of treatment)	II

In one study (1/9), CT cavernosography was used for diagnostic purposes only. Kawanishi et al. found CT cavernosography to be superior to conventional cavernosography for site-specific venous leak identification (100% vs. 19.4%, respectively) [26]. In this study, conventional cavernosography images were simulated using maximum intensity projection images of CT.

Eight studies (8/9) include both diagnostic imaging and treatment planning of venous leak in patients with venogenic ED [11, 19, 25, 27–31]. If penile duplex sonography is positive for venogenic ED, CT cavernosography should be performed for confirmation of venous leak including its morphological presentation for treatment planning [11]. Of interest, Sussman et al. reported low negative predictive values (47%) of duplex sonography for diagnosing venous leak compared to CT cavernosography [29].

Three studies (3/9) describe anatomical venous leak classifications that were established based on CT cavernosography findings [11, 19, 27]. These classifications allow for differentiation of superficial and/or deep venous leak. Mixed or more complex forms of venous leak were present in up to 84% of patients demonstrating the value of CT cavernosography for treatment planning [11]. CT cavernosography enabled accurate mapping of venous leak and smaller caliber collateral veins, thus reducing the risk of treatment failure [19, 27].

In one study (1/9), venous leak embolization was performed using particularly antegrade access via a deep dorsal penile vein or a transfemoral venous approach (primary clinical success rate 68%) [31]. Another two studies (2/9) used venous sclerotherapy [28, 30]. Allaire et al. report an hybrid treatment approach combining surgery with endovascular venous embolization with a primary clinical success rate of 73% at 14 months post treatment. Of interest, they were using CT cavernosography not only for work-up, but also for follow-up.

## Discussion

### Management algorithm

Various studies have demonstrated that, if venous leak is suspected on penile duplex sonography, CT cavernosography should be performed to confirm the venous leak and to obtain site-specific information on the leak as well as its classification [11, 19, 25–36]. However, there remain some inconsistencies in the current literature. Some recently published studies still refer to dynamic infusion cavernosometry and cavernosography as the gold standard for diagnosing venogenic ED, and CT cavernosography was not considered [2, 37–39]. This stands in contrast to other studies where cavernosometry is no longer part of the diagnostic algorithm and cavernosography was replaced by CT cavernosography [25, 27, 29]. Furthermore, CT cavernosography is also not yet mentioned within the American Urological Association (AUA) guidelines and European Association of Urology (EAU) guidelines [40, 41].

### Anatomical classifications

The use of CT cavernosography for diagnosis and treatment planning was first reported by Ghafoori et al. [25]. The authors concluded that cross-sectional CT cavernosography was able to delineate multiple leaking veins in anatomical detail in patients with venogenic ED. With conventional cavernosography, veins may frequently overlap other veins, bony structures or the cavernous body, which may inevitably lead to fewer diagnostic images, and the origin of a venous leak may remain unknown [19]. Kawanishi et al. reported that 80% of patients with a suspected venous leak of a deep dorsal vein on conventional cavernosography did not actually have a leak of this vein but rather a leak of another vein on CT cavernosography [26]. In these patients with venogenic ED, CT cavernosography enabled a site-specific diagnosis of venous leak with precise anatomical depiction of penile and pelvic veins without being influenced by disruptive superimposing structures. Xu et al. could also demonstrate that CT cavernosography provides more detailed anatomical information on venous leak than conventional cavernosography [19]. CT cavernosography depicts both, anatomical and pathological structures, very accurately enabling a more detailed classification of venous leak. CT cavernosography allows for the classification of venogenic ED into six types according to the location of the venous leak: (A) superficial venous leak, (B) deep venous leak, (C) crural venous leak, (D) cavernosal venous leak (E) venous leak between the penis and urethra cavernosum, and (F) mixed venous leak.

Another classification of the deep dorsal vein, prostatic venous plexus and crural vein was described by Ye et al. [11]. This classification consists of three groups: superficial veins, intermediate veins and deep veins and was used for general assessment in their study. Furthermore, different types of origins could be determined for deep dorsal veins, prostatic venous plexus, and crural veins. The authors conclude that using CT cavernosography different types of deep dorsal vein and prostatic venous plexus (single branch, multiple branches, or complex) were distinguished and crural vein origins mapped.

Virag et al. proposed classifying venogenic ED into 4 groups based on CT cavernosography findings: (A) no drainage, (B) drainage via deep veins and respective branches, (C) drainage via superficial veins, and (D) drainage via both deep and superficial veins [27]. Using this classification, previous penile venous surgery failures were identified and further explained, e.g., persistent erectile dysfunction after isolated ligation of a deep dorsal vein due to newly formed collateral veins or superficial draining veins that may have been previously neglected. This additional knowledge appears to be highly beneficial for the surgical treatment of venogenic ED.

### Treatment planning

To date the level of evidence for recommendations regarding penile vein ligation appears to be low and long-term success rates of surgical penile vein ligation have been reported to be approximately 25% [42]. There are still unresolved controversies and universal diagnostic criteria for patient selection and surgical technique have not been clearly established. This type of surgery may be offered in special situations or under study conditions using pre- and post-operative validated outcome measures. Nevertheless, it should be noted that in special cases of venous leak, e.g., due to congenital or post-traumatic lesions, targeted surgical ligation of venous leak may be appropriate. Therefore, understanding both venous anatomy and the underlying pathology is essential for therapeutic success [25–27, 29, 32].

**Fig. 3:**
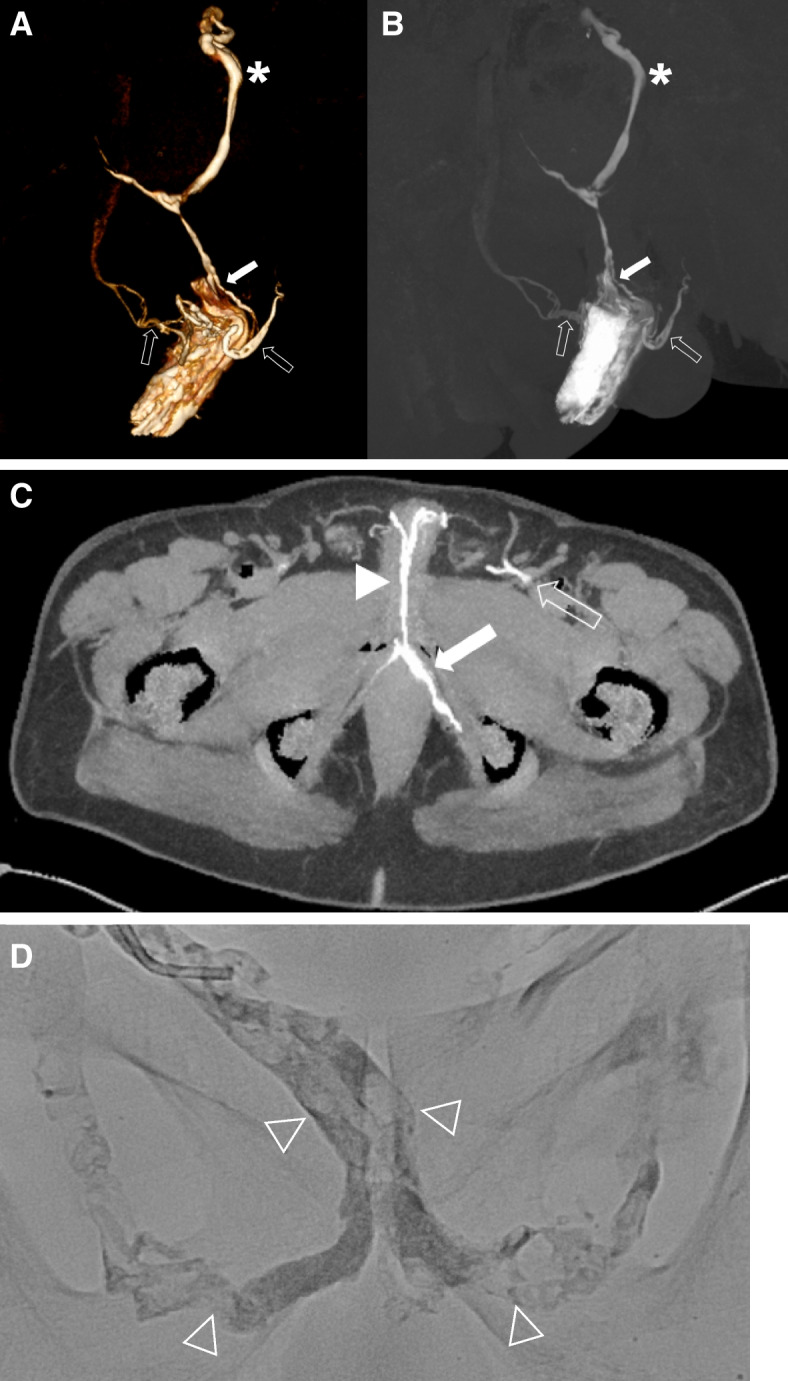
A 58-year-old patient with severe erectile dysfunction not responding to phosphodiesterase-5-inhibitors. CT-cavernosography depicting venous leaks of the deep dorsal penile vein (arrowhead), internal pudendal veins (arrows) draining into iliohypogastric veins (asterisk) and external (open arrows) pudendal veins on three-dimensional volume rendering (**A**), three-dimensional maximum intensity projection (**B**), and axial maximum intensity projection images (**C**). Image post venous leak embolization (**D**) with NBCA-based liquid embolic agent mixed with lipiodol (open arrowheads)

As with surgery, the endovascular treatment strategy is venous leak occlusion. For venous leak embolization n-butyl cyanoacrylate (NBCA)-based liquid embolic agent mixed with lipiodol was used for safe and efficacious embolization with a clinical success rate of 68% (Fig. 3) [31]. Not only the access route, but also the catheter tip position and target veins for embolization are determined depending on the results of pre-interventional CT cavernosography. Pre-interventional it is important to distinguish superficial and deep venous leak on treatment planning such as either embolization with glue for deep venous leak or venous sclerotherapy with foam for superficial venous leak should be performed. Furthermore, possible sites of non-target embolization should be identified on CT cavernosography and protective embolization with coils should be considered on treatment planning. CT cavernosography appears to be more adequate than conventional cavernosography for selecting adequate patients who are likely to benefit from interventional therapy.

### Technical aspects

A multislice CT scanner has the capability of rapidly scanning a large longitudinal volume with high spatial and z-axis resolutions, resulting in a more detailed depiction of anatomical structures on two- and three-dimensional reconstructed images. CT cavernosography is performed after an intracavernosal injection of 10–20 µg alprostadil [19]. After 10–20 min, a 23-G needle is inserted into a dorso-lateral side of the corpora cavernosum. Subsequently, normal saline is gradually injected into the corpora cavernosum using a power injector with increasing flow rates starting at 0.1 ml/s. Once penile tumescence is reached, 10–20 cc of diluted saline nonionic iodinated contrast agent is injected at the previously determined infusion velocity using a power injector. CT images are acquired using a state-of-the-art CT scanner with 80 × 0.5 mm collimation and 0.35 s gantry rotation time. It is a continuous scan with real-time monitoring of venous contrast distribution, starting from the upper brim of the true pelvis to the most distant level of the penis. For postprocessing multiplanar reconstructions (MPRs) in the axial, coronal and sagittal planes, maximum intensity projection (MIP) in two and three dimensions and three-dimensional volume rendering were applied to obtain diagnostic images of venous contrast distribution (Fig. 3).

Both conventional cavernosography and CT cavernosography are able to depict larger anatomical regions of penile and pelvic veins and can therefore be used for diagnostic imaging in ED patients with a suspected venous leak, as previously reported [11]. Compared to conventional cavernosography, CT cavernosography is a cross-sectional imaging method [19]. Its examination time is shorter and less contrast medium is necessary. The average radiation dose is 6.5 mSv (range 1.4 mSv) for conventional cavernosography and 4.5 mSv (range 1.1 mSv) for CT cavernosography. The average amount of contrast agent was 80.6 ml (range 11.5 ml) for conventional cavernosography and 66.5 ml (range 10.1 ml) for CT cavernosography. The average examination time is 577 s (range 148 s) for conventional cavernosography and 481 s (range 156 s) for CT cavernosography.

### Limitations

The potential side effects of CT cavernosography are quite similar to those of cavernosography [19]. Both methods may cause contrast allergic reaction, renal insufficiency, foreskin hematoma, and leak of contrast agent to the skin. Furthermore, dizziness, palpation, sweating and hot flashes may occasionally occur, most likely due to a previous injection of vasoactive drugs. In a few patients, priapism occurred and had to be treated. Otherwise, minor side effects were treated with medical therapy whenever necessary. Occasionally, patient movement during the exam may disturb the accuracy of image acquisition. Kawanishi et al. reported that organs related to erectile function, including the neurovascular bundle, internal pudendal artery, accessory pudendal artery, and corpora cavernosa, may be exposed to radiation during (CT) cavernosography. Although previous studies found that this risk was not highly relevant, efforts should still be made to reduce radiation exposure as much as possible, and patients’ gonads may be protected using a cylindrical protector [26]. Finally, it is not possible to distinguish histological abnormalities in patients with corporal veno-occlusive dysfunction, such as corporal fibrosis or a decrease in smooth muscle cells. Further research is advised to fully elaborate both the benefits and potential drawbacks of CT cavernosography.

### Other applications

Izumi et al. described virtual cavernoscopy reconstructing virtual cavernoscopic images from contrast enhanced CT datasets of ED patients [43]. Using different window settings, virtual cavernoscopy was able to visualize the lumen of the corpora cavernosa, venous outlets, and cavernous arteries. However, the additional clinical benefit of this imaging modality needs to be further elaborated.

CT cavernosography was also used for evaluating functional penile anomalies in patients with Peyronie's disease [44]. A pilot study assessed images of degree of penile curvature, presence of corporal involvement, and location of corporal involvement. The authors concluded that CT cavernosography may provide additional information on penile anatomy in men with Peyronie's disease that is not provided by existing methods of evaluation. However, studies with a larger patient cohort and a longer clinical follow-up are required.

Of interest, magnetic resonance imaging (MRI) was used for visualization of venous leaks in patients with venogenic erectile dysfunction [45, 46]. These studies confirmed that MRI may be suitable as a diagnostic tool for assessing venous leaks in ED patients. Compared with (CT) cavernosography, this method does not cause radiation exposure to the patient. However, the availability of MRI may be limited, and specialized medical personnel and materials are required for intracavernosal contrast administration during the procedure. Furthermore, examination times may be significantly longer than those of CT cavernosography. Additional studies are needed to further compare MRI with cavernosometry, cavernosography, and especially CT cavernosography.

## Conclusion

CT cavernosography allows for site-specific identification of venous leak in ED patients for diagnosis and treatment planning (grade II levels of evidence). In comparison to conventional cavernosography, CT cavernosography is faster to perform, exposes the patients to less radiation and requires less contrast material. Moreover, CT cavernosography should be included in management algorithms for ED patients with suspected venous leak.

## Data Availability

Patient data and materials are available.

## Abbreviations

AUA: American Urological Association
CT: Computed tomography
CTA: Computed tomography angiography
EAU: European Association of Urology
ED: Erectile dysfunction
EDV: End-diastolic velocity
MIP: Maximum intensity projection
MPR: Multiplanar reconstruction
MRI: Magnetic tomography imaging
NBCA: N-butyl cyanoacrylate
PDE5i: Phosphodiesterase-5 inhibitors
PRISMA: Preferred Reporting Items for Systematic reviews and Meta-Analyses
PSV: Peak systolic velocity
